# Outpatient and Inpatient Services Satisfaction in Iranian Military Hospitals

**DOI:** 10.5812/ircmj.12665

**Published:** 2013-09-05

**Authors:** Ahmad Ameryoun, Gholamhossein Pourtaghi, Emad Yahaghi, Somaie Heidari, Mohamadkarim Bahadori, Mehdi Ebrahimnia, Kayghobad Tahernezhad

**Affiliations:** 1 Health Management Research Center, Baqiyatallah University of Medical Sciences, Tehran, IR Iran; 2 Department of Management of Health Care, School of Public Health, Baqiyatallah University of Medical Sciences, Tehran, IR Iran; 3 Health Research Center, Baqiyatallah University of Medical Sciences, Tehran, IR Iran; 4 Young Researchers and Elite Club, Damghan Branch, Islamic Azad University, Damghan, IR Iran

**Keywords:** Patient Satisfaction, Clinic, Hospital, Ambulatory Care, Health Services

## Abstract

**Background:**

Many diagnostic and treatment procedures are done in hospitals and clinics. Offering services in these areas have a prominent role in promoting patients’ satisfaction levels and their prospective about health services.

**Objectives:**

This study is going to assess the satisfaction levels of patients referring to the six military hospital clinics in Iran.

**Materials and Methods:**

In this cross-sectional study, 330 outpatients and 696 inpatients admitted to the six military hospital clinics in Iran were randomly questionnaires from June to August 2008. Basic socio-demographic data along with a clinic satisfaction level assessment questionnaire were filled for outpatients. A hospital satisfaction level assessment questionnaire also was applied to record inpatients’ data. All collected data were recorded and then analyzed tests X2 and ANOVAs was used and with significantly lower levels of 5% (P < 0.005).

**Results:**

We found that 96% of the study population was satisfied with clinic services and more than 98% of the respondents were satisfied with inpatient ward services. In clinic services, the satisfaction level in numbering and waiting time, access to the clinic, physical environment, welfare and helping facilities, and personnel and physicians’ behavior were 78.2%, 80.6%, 89.1%, 91.2% and 93.6% respectively (P < 0.001). With regard to inpatient services, the satisfaction level of patients with physician services, nursing routine services, behavior of nurses, nutritional condition, welfare facilities, reception unit services, discharge unit services and accounting unit services were 94.7%, 91.9%, 91.9%, 91.5%, 91.5%, 91.2%, 90.8% and 88.2%, respectively (P = 0.013).

**Conclusions:**

On the basis of the findings, most respondents reported having a favorable satisfaction with clinic and hospital health services. However, planning to reduce patient’s waiting time in clinics and training physicians to offer more instructions to the patients seems necessary. Since discharge and accounting unit services had the lowest satisfaction levels of inpatients services, responsible managers must have special attention to these official processes.

## 1. Background

Attention to the health issue reveals clearly that quantitative aspect of health services was the priority of health services programs in the past (1). Today, however, assessment and realizing the deficits and the quality of accessibility to the optimum health services are the major concerns of the policy makers in health worldwide (2). Therefore, managers and planners of health care systems today are much more interested in improvement of quality of cares (3). It is clear that the assessment of current condition and planning for further improvement, is quite necessary to promote the services (4). One of the major factors affecting quality of health care services is the patients’ satisfaction level. It can provide us a better understanding of strengths, weaknesses and solvable problems from patients prospective (3, 5). Hence, assessment of patients’ satisfaction level is now one of the five WHO indicators to improve the quality of health care services (6, 7). Researchers believe that this factor is one of the most important factors determining the level of health services (8). Numerous studies have shown that a greater satisfaction level of patients with physicians’ behavior and their consultations during the treatment period encourage them to follow physician recommendations and orders better (9). Establishment of a more profound relation between patient and health care personnel would lead to a better follow of drug regimens and treatment advices. It also can help to achieve better health outcomes due to good satisfaction level of patients (10, 11). Since patients’ satisfaction level is an important index of quality of health services in various domains such as interpersonal, organizational and technical domains, its assessment provides an important information source to detect problems and present ideal health care services too (12). One of the commonest study groups in this field is hospitalized patients. Patients’ prospective regarding quality of hospital health care services is an important issue in the assessment of satisfaction level of health services (10, 13). It is shown that patients’ satisfaction with hospital health services is affected by various factors such as physician, nurses and other personnel functions and physical environment of the hospital (14, 15). It can also predict future behaviors of patients during treatment course and after discharge. On the other hand, clinics are always considered as the first contact place between patients and health care system (16). Since a great deal of diagnostic procedures, treatment approaches and follow-up of chronic diseases are delivered in the clinics, many patients tend to attend to this part of health system. Therefore, the assessment of satisfaction level of these patients would provide a chance to evaluate of non-visible aspects of services such as waiting time, to prevent from wasting sources and finally to reduce the costs of health care services (17). Numerous studies have been done on assessment of patients’ satisfaction level with health services in our country, mostly in one hospital or clinic in a certain city and therefore reflect viewpoints of inhabitants of that specific geographical territory (4, 6, 18-25).

## 2. Objectives

This study, however, was done on assessment of the satisfaction level of inpatients and outpatients of six military hospitals and their clinics regarding health services throughout the country. The assessment process should be monitored regularly to the satisfaction of the management approach for analysis and remedy the causes of the changes.

## 3. Materials and Methods

We carried out a cross-sectional study from July to September 2008 to assess patients’ satisfaction levels with health system. Study population, a total of 1026 patients, was randomly selected from inpatients of six military hospitals and outpatients referring to their clinics in six large cities throughout the country, Iran. 55 patients from each clinic and 116 patients from the wards of each hospital were selected (330 outpatients and 696 inpatients). Wards selected from different parts of the hospital. All patients were 15 year-old or older and were psychologically healthy. The questionnaire was completed by patients after discharge location. National standardized questionnaire was used. The pilot phase was also examined and its validity was confirmed with 90% confidence. Outpatients were questionnaires following completion of official and treatment processes and inpatients were questionnaires after discharge from the hospital. After introducing, questionnaires described the aims of the study to the patients and ask them to participate in the study. Cities in military hospitals and the general public have been selected. The sample size were based on Cochran formula. Reported actual P values for primary analyses is P = 0.003 and P = 0.001. Inclusion criteria for this study have at least 15 years of age, having the physical and mental fitness Webster was in hospital for at least 24 hours. Every question once of each person and there was no duplication. About Critically patients in accordance with the discretion of the supervisor survey, information was not collected. A psychiatric ward because of the uncertainty of the existence of critically patients, the study excluded patients adhere to ethical issues and lack of comfort away. An anonymous checklist containing demographic information including age, gender, marital status and education levels, insurance and previous admission to that hospital or clinic was completed for every patient. Then a clinic satisfaction level questionnaire for outpatients and the hospital satisfaction level questionnaire for inpatients were completed. The clinic satisfaction level questionnaire had 24 items and assessed patients’ satisfaction level with clinic health services on following domains; numbering and waiting time (4 items), accessibility to the clinic (1 item), physical environment, welfare and helping facilities (6 items), behavior of personnel (3 items) and health services delivered by physicians (10 items). This physician services divided into the three domains: behavior and observation of patients’ rights and their religious customs (4 items), sufficient knowledge and rapid examination (4 items) and offering instructions to the patients about laboratory and radiologic findings and future follow-up (2 items). The hospital satisfaction level questionnaire had 79 items and assessed hospital health services in the 8 domains: physician services (10 items), nursing routine services (12 items), behavior of nurses (15 items), nutritional condition (11 items), Physical environment and welfare facilities (14 items), reception unit services (6 items), discharge unit services (7 items) and accounting unit services (4 items). A three-choice Likert scale (dissatisfied, not satisfied nor dissatisfied and satisfied) with a 1 to 3 point for each option, respectively, was designed to assess the subjects’ responses. The average of sum of all items in each questionnaire formed the total satisfaction point. The score of 2 or less considered as dissatisfaction and the score higher than 2 considered as satisfaction. SPSS 13 for Windows was used for statistical analysis. Frequency and relative frequency (percent) used for description of qualitative variables. The Chi-square test was utilized for comparison among various parts of hospital and clinic health services. P value less than 0.05 was considered as significant level. Compliance with ethical considerations is: 1. referred to hospitals across the country With reference to official bodies. 2. The characteristics of the patients in this study is voluntary individual will remain confidential. 3. The results will be provided to the relevant authorities. 4. Results of the study will be reflected to the hospital.

## 4. Results

In outpatients group, there were 185 male (56%) and 220 married (67%). Majority of them lay in aged group less than 25 years (127 people, 38.5%). There were 348 male (50%) and 564 married (81%) in inpatients group. 29% of them lay in age group more than 45 years (202 people). Other demographic data are shown in Table 1. 

**Table 1.** Frequency Distribution of Demographic and More Important Characteristics in the Study Population

Variable	Inpatients Frequency (%)	Outpatients Frequency (%)
**Sex**		
Male	348 (50%)	185 (56%)
**Age (year)**		
< 25	195 (28%)	127 (38%)
25 - 35	181 (26%)	109 (33%)
36 - 45	118 (17%)	59 (18%)
> 45	202 (29%)	35 (11%)
**Education level**		
High school and Lower	550 (79%)	220 (67%)
**Marital status**		
Married	564 (81%)	220 (67%)
**Insurance**	675 (97%)	318 (96%)
**Previous admission to the Study hospital/Clinic**	376 (54%)	233 (71%)

**Table 2.** Satisfaction Level of Outpatients From Different Parts of Clinic’s Health Services

	Satisfied	Dissatisfied	P value ^a^
**Clinic’s health services**			< 0.001
Physician	309 (93.6%)	21 (6.4%)	
Behavior of personnel	301 (91.2%)	29 (8.8%)	
Physical environment, welfare and helping facilities	294 (89.1%)	36 (10.9%)	
Accessibility to the clinic	266 (80.6%)	64 (19.4%)	
Numbering and waiting time	258 (78.2%)	72 (21.8%)	
**Health services offered by physicians**			0.003
Behavior and observation of patients’ rights and their religious customs	301 (91.2%)	29 (8.8%)	
Sufficient knowledge and rapid examination	299 (90.6%)	31 (9.4%)	
Offering guidelines to the patients about laboratory and radiologic findings and future follow-up	276 (83.6%)	54 (16.4%)	

^a^ Chi-Square Test

**Table 3.** Satisfaction Level of Inpatients From Different Parts of Hospital Health Services

	Satisfied	Dissatisfied	P value ^a^
**Physicians**	659 (94.7%)	37 (5.3%)	0.013
**Nursing routine services**	640 (91.9%)	56 (8.1%)	
**Behavior of nurses**	640 (91.9%)	56 (8.1%)	
**Nutritional condition**	637 (91.5%)	59 (8.5%)	
**Physical environment and welfare facilities**	637 (91.5%)	59 (8.5%)	
**Reception unit services**	635 (91.2%)	61 (8.8%)	
**Discharge unit services**	632 (90.8%)	64 (9.2%)	
**Accounting unit services**	614 (88.2%)	82 (11.8%)	

^a^ Chi-Square Test

Generally, more than 95% of the participants were satisfied with clinics health services. As shown in Table 2, health services offered by physicians had the highest satisfaction level (93.6%) and numbering and waiting time with 78.2%, received the least satisfaction level (P < 0.001). In the field of physician services, physician behavior and observation of patients’ rights had the highest satisfaction level (91.2%). Presenting instructions to the patients regarding laboratory and radiologic findings and future follow-up with 83.6%, however, received the lowest satisfaction level (P = 0.003). With regards to inpatients and hospital satisfaction, generally, 98.2% of the subjects were satisfied with hospital health services. In this field, services offered by physicians with 94.7% and accounting unit services with 88.2% received the highest and the lowest satisfaction levels, respectively (P = 0.013). Table 3 shows satisfaction levels of other parts of inpatient health services. 

## 5. Discussion

On the basis of the findings a great deal of patients referred to the studied hospitals and related clinics included in this study had a favorable satisfaction with health services. Generally, hospital health services had a higher satisfaction level than other domains. Among different domains of services, the physicians’ services in clinics and hospitals received the highest satisfaction rate. Offering instructions to the patients by clinics’ physicians, however, got the lowest satisfaction level. The numbering and waiting time in clinic, health services, discharge and accounting unit services in hospitals had the low satisfaction levels, too. Although there are great differences in findings of similar studies in our country, generally, patients’ satisfaction level with health services is somehow favorable. However, findings of this study reveal more satisfaction level with inpatient and outpatient wards services. While the satisfaction level of clinics in our county has been reported 70% to 80% (6, 18), this rate was more than 95% in the current study. Overall patients’ satisfaction level with hospital health services was more than 98% while satisfaction level in similar studies was significantly different and ranged from 50% to 95% (20-24). Numerous studies have shown that satisfaction with physicians has important role in the overall patients’ satisfaction level (26). That is, Patients who are more satisfied with physicians consider their advices and orders more seriously, and have better treatment outcome (9, 15). Factors such as on-time presence of physician, exact examination and proposing description about disease nature and its future follow-up have an important role in this field (16, 29, 30). Satisfaction level of more than 90% with clinic physicians in our study showed a favorable contentment compared to the similar studies (6, 16, 18). However, as mentioned earlier, offering instructions by physicians had the lowest satisfaction level among services offered which needs more attention. Because of high rate of referring patients to the clinic, it may be impossible to get complementary information about treatment and follow-up by physician. Informing patients regarding future visits and disease follow-up by other health personnel has an important role in reducing patients’ stress and ambiguity. It can, in turn, improve quality of health care services and patients’ satisfaction level. The services offered by hospital physicians had the highest satisfaction level, too, which was much greater than other similar studies (23-25). One of the domains which usually has great dissatisfaction rate is numbering queue and waiting time in clinics. In our study more than 21% of study population had different levels of dissatisfaction with this item which is lower than what is been reported via similar previous studies (18). Another common health system problem is prolonged waiting time to get outpatient health services (19, 31). This long-lasting time resists offering favorable health services, wastes patients’ time and dissatisfies them. Therefore, Patients’ satisfaction with waiting time has a prominent role in the quality assurance of health services and its management (19). The survey was carried out by Mahmoud Mousa Zadeh et al. Assigned to the meta-analysis showed that in patients admitted to the hospital Percent of hospital inpatient satisfaction considering the heterogeneity of Iran , based on the random effect model 70/5 is estimated. Although patient satisfaction in this model is higher than the fixed effect model. However, due to the heterogeneity of the studies reported in this indicator is based on a random effects model is approved. Although patient satisfaction is higher than average. This number means that in 30% of patients were dissatisfied with the services provided by hospital contact the hospital as one of the most important components of the health system, not a pleasant experience is desirable Due to legal restrictions advertising for healthcare organizations, today's leading hospitals around the world as one of the most important tools of the patients are given verbal advertising. Hence, there is 30% dissatisfaction with hospital care in terms of oral advertising; the hospital does not transcend the desired image.

However, the percentage of patient satisfaction compared with other countries in the situation is favorable. Case studies of patient satisfaction 52% in America, 90% in Britain, 58% in South Korea, 91/7%, Canada , 70% in Tunisia and 76 %in Pakistan over the report (32). Because of high rate of referring patients to get outpatients health services, usually patients wait for a long time, so using welfare facilities such as television, video training films, and etc. in this period can improve patients’ health knowledge and help them to spend this time more convenience. In addition early scheduled appointments might be helpful in reducing the waiting time. Considering the findings of hospitalized patients showed that overall satisfaction level of patients can be divided into two separate sections. First, is related to the health services throughout hospitalization, following admission until discharge, and the second, is related to the accessory services such as reception, discharge, and accounting units’ services. The results of these sections reveal apparently that patients’ satisfaction level with first section was better than the second. Although, compared to previous similar studies, our findings show a higher satisfaction level in most domains of inpatient health services, a higher dissatisfaction level with official process such as discharge and accounting units had mentioned by other studies too (20, 23). It seems that this part of health care services is one of the major concerns of our patients and therefore needs more attention by health authorities and managers. As disease stress may lead to decrease the patient and his/her family tolerance and official services are time consuming process, therefore, changing health official rules which decreases the level of involvement of patients in reception and discharge may help in resolving this problem. The present study was one of the scant studies assessing patients’ satisfaction levels with clinic and hospital services throughout of our country. Satisfaction in this study was higher than in other studies because type of hospitals and free services hospitals and insurance coverage, Achieved a higher satisfaction rate than normal. Although assessment of a great deal of inpatients and outpatients from various parts of our country was one of the prominent aspects of our study, not considering disease types and patients’ treatment outcomes or relieving their needs were the limitations of this study. Hence, establishment of a process for continuous assessment of patients’ satisfaction levels for detecting of weak points of health systems and planning for mending them is recommended to promote health of the society.
